# Mitochondrial DNA association study of type 2 diabetes with or without ischemic stroke in Taiwan

**DOI:** 10.1186/1756-0500-7-223

**Published:** 2014-04-09

**Authors:** Jun-Hun Loo, Jean A Trejaut, Ju-Chen Yen, Zong-Sian Chen, Wai-Mei Ng, Chin-Yuan Huang, Kuang-Nan Hsu, Kuo-Hua Hung, Yachun Hsiao, Yau-Huei Wei, Marie Lin

**Affiliations:** 1 Mackay Memorial Hospital, No. 45, Mínshēng Rd, Danshui District, New Taipei City, Taiwan; 2 Mackay Memorial Hospital, Taitung branch, Lane 303, Changsha Street, Taitung City, Taiwan; 3 Mackay Memorial Hospital, HsinChu branch, #690, Section 2, Guangfu Road, Hsinchu City, Taiwan; 4 National Yang-Ming University, Beitou District, 112, Taipei City, Taiwan; 5 Mackay Medical College, New Taipei, Taiwan

## Abstract

**Background:**

The importance of mitochondrial DNA (mtDNA) polymorphism in the prediction of type 2 diabetes (T2D) in men and women is not well understood. We questioned whether mtDNA polymorphism, mitochondrial functions, age and gender influenced the occurrence of T2D with or without ischemic stroke (IS).

**Methods:**

We first designed a matched case–control study of 373 T2D patients and 327 healthy unrelated individuals without history of IS. MtDNA haplogroups were determined on all participants using sequencing of the control region and relevant SNPs from the coding region. Mitochondria functional tests, systemic biochemical measurements and complete genomic mtDNA sequencing were further determined on 239 participants (73 healthy controls, 33 T2D with IS, 70 T2D only and 63 IS patients without T2D).

**Results:**

MtDNA haplogroups B4a1a, and E2b1 showed significant association with T2D (P <0.05), and haplogroup D4 indicated resistance (P <0.05). Mitochondrial and systemic functional tests showed significantly less variance within groups bearing the same mtDNA haplotypes. There was a pronounced male excess among all T2D patients and prevalence of IS was seen only in the older population. Finally, nucleotide variant np 15746, a determinant of haplogroup G3 seen in Japanese and of B4a1a prevalent in Taiwanese was associated with T2D in both populations.

**Conclusions:**

Men appeared more susceptible to T2D than women. Although the significant association of B4a1a and E2b1 with T2D ceased when corrected for multiple testings, these haplogroups are seen only among Taiwan Aborigines, Southeast Asian and the Pacific Ocean islanders where T2D is predominant. The data further suggested that physiological and biochemical measurements were influenced by the mtDNA genetic profile of the individual. More understanding of the function of the mitochondrion in the development of T2D might indicate ways of influencing the early course of the disease.

## Background

Mitochondrial DNA (mtDNA) codes for thirteen protein subunits of the mitochondria electron transport chain (ETC.), two ribosomal RNAs and twenty-two transfer RNAs that are essential in the mitochondrial protein synthesis. Although the complete mtDNA genome size is very small compared to nuclear DNA, its high mutation rate (28.2 to 42.5 greater than nuclear DNA) suggests that the role of mtDNA holds an important and active role in human evolution [1].

At the end of the Paleolithic period, the ancient continent of Sundaland was being covered with the rise of sea levels and separated Taiwan and Western Island Southeast Asia (ISEA) from the Asian Continent [2]. Relocation and ensuing long isolation of its peoples resulted into the emergence of several mtDNA sub-haplogroups (HG) specific to Taiwan, ISEA and Oceania such as B4a1a and E and rarely seen in continental Asia [2,3]. The weather in Taiwan, ISEA and Oceania is generally hot, humid. This climate not only provides an ideal environment for infective microorganisms to develop, but must have also acted on the populations who adapted to their new environment and with the effect of drift have lead to the expansion of mtDNA profiles unique to the Austronesian speakers of Taiwan and ISEA [4,5].

While the economic status in Taiwan and ISEA after World War II allowed for the introduction of antibiotics, a significant decrease in infective diseases and a rapid improvement of hygiene, the Western type of food and a sedentary life style greatly contributed to obesity, and prevalence of type 2 Diabetes (T2D). The number of people with T2D in the world is close to 240 million, with East Asians being the most affected and their number expected to continue increasing if no effective preventive steps are taken. Interestingly, it has also been hypothesized that while insulin resistance favor T2D, it may have also provided a more efficient way to use energy to individuals in their new environment and therefore a more efficient way to fight infection [6]. It was later reported that the presence of mtDNA nucleotide position (np) 15746 in Japanese and Korean people [7-9] may be associated with T2D (or T2D with Ischemic Stroke (T2D^IS^)). Since the mtDNA profile of the Japanese is significantly different to the Taiwanese we aimed at determining whether np 15746 was actually an inherited ancestral trait associated to T2D, or the recurrence of a mutation occurring in distinct mtDNA haplogroups associated to T2D in different populations. Further, we analyzed extensively the relationship between T2D and control groups, their mtDNA polymorphism and their mitochondria biological functions in Southern Taiwan.

## Methods

Unrelated individuals (N = 808) over 45 years old from the Mackay Memorial Hospital Taitung branch in southeastern Taiwan were recruited for this study. T2D is usually associated with vascular complications (i.e. Ischemic stroke, myocardial-infraction, diabetic retinopathy, nephropathy, small-vessel disease, large-artery atherosclerosis, etc.). A substantial proportion of T2D cases in our sampling had also history of ischemic stroke (17.43%) and were included in the analysis and Ischemic stroke patients with no T2D history were later recruited for comparison. Finally, the data set comprised 327 unrelated healthy individuals (controls), 308 patients with T2D only (T2D^only^), 65 patients with T2D and history of ischemic stroke (T2D^IS^), and 108 patients with IS only (IS^only^). Indication on overnight fasting glucose, glycohemoglobin level (HbA1c), kidney function, and medication records of cases and control groups are listed in Additional file 1: Table S1. All individuals provided their name, birthday, parent ethnic affiliation, filled an epidemiological questionnaire and gave informed consent to participate in the study. The project was approved by the ethics committee of Mackay Memorial Hospital, Taiwan.

The following measurements and tests were conducted at the Mackay Memorial Hospital Taitung branch using standard techniques:

*Individual’s physical outlook*: height, weight, body mass index (BMI), waist circumference.

*Physiological measurements*: systolic blood pressure, diastolic blood pressure.

*Blood biochemistry measurements*: Overnight fasting glucose, High Density Lipoprotein (HDL), Low Density Lipoprotein (LDL), total cholesterol (TC), triglyceride, uric acid, creatinine.

### MtDNA sequencing

To analyze the polymorphism of mtDNA, DNA was extracted from 500 μl of buffy coat from each blood sample using the QIAmp DNA kit (QIAmp® DNA Blood Mini kit from Qiagen inc. Taiwan). For mtDNA typing, control region HVS-1, nucleotide positions (np) of coding region fragments 8000 to 9000 and 9800 to 10900 were sequenced using a method previously publications described [3,10]. Complete mtDNA genome sequencing was carried out on a total of 239 individuals (73 controls, 70 T2D^only^, 33 T2D^IS^, and 63 IS^only^). Briefly, 24 fragments of mtDNA were amplified and sequenced in both directions [11]. Haplogroup assignments were done according to the “phylotree” criterion (http://www.phylotree.org) using combinations of the HVS-1 sequence, partial sequencing of the coding region, and other relevant diagnostic variants. Each individual were assigned to an ethnic group (either Taiwan non-Aborigine or Taiwan Aborigine) according to the epidemiological questionnaire which was filled at the time of blood sample collection.

### Mtochondrial functional study

Under inflammation, cellular mitochondria develop membrane hyperpolarization. These morphological changes are also seen in the mononuclear cells of T2D patients [12]. Venous ACD blood collection in BD Vacutainer® (Becton Dickinson, USA) and stored at 4°C for less than 24 hours was found to be the best condition to transport specimens to Taipei (350 km) for measurements of mitochondrial functions and antioxidant capacities of the mononuclear cells.

### Isolation of mononuclear cells from peripheral blood

After centrifugation of the venous blood at 3000 g for 10 min, the buffy coat (2 mls) was diluted with an equal volume of phosphate buffered saline solution (PBS), layered over 2 ml Ficoll-Paque PLUS (GE Healthcare Bio-Sciences AB, Sweden) and centrifuged at 2000 g for 20 min to separate mononuclear cells from polymorphonuclear neutrophils. The mononuclear cells were removed and washed twice with PBS, and suspended in RPMI 1640 medium (GIBCO, USA) until used. The viability of all mononuclear cells, measured by trypan blue dye exclusion, was greater than 95%. Due to the small volume collected and the large number of clinical measurements performed for each participant in the study, test were done without replication. Instead, an internal working standard was introduced with each set of experiment. Except for the test of mtDNA copy number, Coefficient of Variation (CV) shown in Additional file 2: Table S2 are inter-assay CV.

### Measurement of ATP levels in mononuclear cells

Intracellular ATP levels were determined using the Bioluminescent Somatic Cell Assay Kit (Sigma-Aldrich, USA) according to the manufacturer’s instructions.

### Measurement of hydrogen peroxide production (DCF) and lipid-peroxidation in human mononuclear cells

Intracellular hydrogen peroxide and lipid-peroxidation levels were measured using fluorescent dye 2′, 7′-dichlorodihydrofluorescein diacetate (DCF, Sigma-Aldrich, USA) and C11-BODIPY^581/591^ (Molecular Probes, the Netherlands) respectively. Both fluorescence measurement of DCF at wavelengths 488/520 nm and C11-BODIPY^581/591^ measured by simultaneous acquisition of the green (520 nm) and red signal (695 nm) were obtained by flow cytometry (Becton Dickinson, USA).

### Flow cytometric determination of mitochondrial membrane potential (DiOC)

Mitochondrial membrane potential was measured using 3,3′-diethyloxacarbocyanine iodide [DiOC_2_(3)] (Molecular Probe, The Netherlands) labeled cells by flow cytometry at 488/520 nm wavelengths.

### Measurement of superoxide dismutase (SOD) and catalase activity in human mononuclear cells

Mononuclear cells were suspended in NEP buffer (150 mM NaCl, 0.5 mM EDTA, 0.1 M LHPO4 buffer and 1% Triton X-100, pH 7.5) containing protease inhibitors (Roche Diagnostics, USA). Cell suspension was incubated on ice for 30 minutes at 4°C and the supernatant collected for measurement of enzyme activity. SOD activity was performed using the superoxide dismutase assay kit (Cayman Chemical, USA) according to the manufacturer’s instruction and catalase activity was determined by monitoring the rate of decomposition of hydrogen peroxide from the decrease in absorbance at 240 nm.

### Measurement of total antioxidant capacity (PAO) of blood serum

The total antioxidant capacity in serum was performed by the use of the PAO kit (Nikken SEIL Co., Japan) according to the manufacturer’s instruction.

### Measurement of 8-hydroxy-2-deoxy guanosine (8OHdG) level in blood serum

The level of 8-OHdG in serum was performed by the use of the 8-OHdG EIA kit (Cayman Chemical, USA) according to the manufacturer’s instruction.

### MtDNA copy number determination

DNA aliquots of 50 ng were subjected to quantitative PCR using FastStart Universal SYBR Green Master (ROX) kit (Roche Applied Science, Switzerland) on 7500 Fast Real-Time PCR System (ABI, USA). Both, DNA fragments of ND1 gene (mtDNA encoded, 5′GGAGTAATCCAGGTCGGT, 5′TGGGTACAATGAGGAGTAGG) and β-actin gene (internal standard, 5′CATGTGCAAGGCCGGCTTC, 5′CTGGGTCATCTTCTCGCGGT) were amplified by PCR using the manufacturer’s protocol. The relative copy number of mtDNA per cell was measured by normalization of the crossing point between ND1 and β-actin using the 7500 Fast System software (ABI, USA).

#### Data analysis

All statistical tests (Chi Squared homogeneity testing, logistic regression, F test, t test, Odds ratio and Fisher exact test) were performed with the use of the SPSS statistical package, version 11.5 (SPSS Inc. Released 2002. SPSS for Windows, Chicago, SPSS Inc.).

Of note, in this study the power of the hypothesis test for B4a1a (or np 15746) in the T2d group reached 0.57, and 0.76 in the T2D group with IS. Because T2D is a multifactor and polygenic disorder, we believe that these powers were satisfactory for the tests.

## Results

### MtDNA polymorphism

Participants were separated into four groups according to their medical history: _Control: Unrelated Healthy individuals with no previous T2D or ischemic Stroke (IS) history

T2D^only^: T2D patients with previous T2D history, but no IS history.

T2D^IS^: T2D patients having previous T2D and IS histories.

IS^only^: Patients with previous IS history but no T2D history.

Comparison of the mtDNA haplogroup frequencies between control and disease groups (Table 1) using Chi Squared homogeneity testing did not shown significant differences (data not shown). On the other hand, when testing for haplogroup pairwise differences between control and disease groups using Fisher exact test, significant differences were seen in the T2D groups for B4c1b2a, D4, E2 (P < 0.01), B4a1a and F4 (P < 0.05), and in the IS group for G (P < 0.05). Of note, no significances were retained when correcting for multiple test.

**Table 1:** Relative and absolute () frequencies and mtDNA haplogroup differences between control and disease groups

**mtDNA haplogroup**	**Control**	**T2D**^**all**^	**T2D**^**only**^	**T2D**^**IS**^	**IS**^**only**^
	**N = 327**	**N = 373**	**N = 308**	**N = 65**	**N = 108**
A	1.83 (6)	2.14 (8)	2.27 (7)	1.54 (1)	2.78 (3)
B4	0.92 (3)	0.54 (2)	0.65 (2)	-	-
B4a	3.98 (13)	1.61 (6)	1.62 (5)	1.54 (1)	-
B4a1a	5.50 (18)	9.12 (34)	7.79 (24)	15.38 (10)*	6.48 (7)
B4a2a	4.59 (15)	1.88 (7)	1.95 (6)	1.54 (1)	3.70 (4)
B4b1a	4.59 (15)	4.29 (16)	4.87 (15)	1.54 (1)	2.78 (3)
B4b1b	0.31 (1)	0.27 (1)	0.32 (1)	-	-
B4b1c1	0.31 (1)	0.54 (2)	0.65 (2)	-	-
B4c1b2a	1.22 (4)	4.83 (18)**	4.22 (13)*	7.69 (5)**	1.85 (2)
B4c1b2b	0.61 (2)	-	-	-	-
B4c1c	0.61 (2)	-	-	-	-
B5	1.22 (4)	0.54 (2)	0.65 (2)	-	0.93 (1)
B5a1	2.45 (8)	1.34 (5)	1.30 (4)	1.54 (1)	-
B5a2	2.14 (7)	4.29 (16)	4.87 (15)	1.54 (1)	-
C	0.61 (2)	1.34 (5)	1.30 (4)	1.54 (1)	-
D	0.61 (2)	0.27 (1)	0.32 (1)	-	-
D4	9.17 (30)	3.49 (13)**	3.25 (10)**	4.62 (3)	10.19 (11)
D5	7.03 (23)	6.17 (23)	6.17 (19)	6.15 (4)	6.48 (7)
E1a	1.22 (4)	2.95 (11)	3.25 (10)	1.54 (1)	2.78 (3)
E1a1a	4.59 (15)	5.09 (19)	5.52 (17)	3.08 (2)	2.78 (3)
E2	0.61 (2)	3.48 (13)**	3.90 (12)**	1.54 (1)	0.93 (1)
F1	0.61 (2)	0.80 (3)	0.65 (2)	1.54 (1)	-
F1a	0.92 (3)	0.80 (3)	0.97 (3)	-	1.85 (2)
F1a1a	2.45 (8)	1.07 (4)	1.30 (4)	-	3.70 (4)
F1a3	2.45 (8)	2.68 (10)	3.25 (10)	-	3.70 (4)
F1a4	0.92 (3)	1.34 (5)	1.30 (4)	1.54 (1)	1.85 (2)
F1a5	0.31 (1)	-	-	-	0.93 (1)
F2	1.83 (6)	1.07 (4)	0.97 (3)	1.54 (1)	2.78 (3)
F3	5.20 (17)	6.17 (23)	5.52 (17)	9.23 (6)	4.63 (5)
F4	2.45 (8)	5.63 (21)*	6.17 (19)*	3.08 (2)	5.56 (6)
G	0.31 (1)	0.80 (3)	0.97 (3)	-	2.78 (3)*
M	0.92 (3)	0.54 (2)	0.65 (2)	-	-
M10	0.61 (2)	0.27 (1)	0.32 (1)	-	-
M12	0.61 (2)	0.54 (2)	0.65 (2)	-	0.93 (1)
M33	0.61 (2)	0.27 (1)	-	1.54 (1)	0.93 (1)
M7	0.92 (3)	0.80 (3)	0.32 (1)	3.08 (2)	-
M7b	-	1.07 (4)	0.97 (3)	1.54 (1)	-
M7b1	5.50 (18)	2.68 (10)	2.60 (8)	3.08 (2)	8.33 (9)
M7b3	1.83 (6)	1.88 (7)	1.95 (6)	1.54 (1)	1.85 (2)
M7b4	0.31 (1)	0.27 (1)	-	1.54 (1)	-
M7c	1.22 (4)	0.54 (2)	0.65 (2)	-	-
M7c1	0.31 (1)	1.34 (5)	1.30 (4)	1.54 (1)	0.93 (1)
M7c2	0.31 (1)	0.27 (1)	0.32 (1)	-	-
M7c3a	2.75 (9)	3.22 (12)	3.57 (11)	1.54 (1)	0.93 (1)
M7c3b	0.31 (1)	0.27 (1)	0.32 (1)	-	-
M7c3c	3.36 (11)	4.56 (17)	4.55 (14)	4.62 (3)	6.48 (7)
M8a	2.14 (7)	1.88 (7)	1.95 (6)	1.54 (1)	2.78 (3)
M9a	0.61 (2)	-	-	-	0.93 (1)
N9a	2.75 (9)	2.41 (9)	1.95 (6)	4.62 (3)	3.70 (4)
R9	2.45 (8)	1.61 (6)	1.62 (5)	1.54 (1)	2.78 (3)
Z3	0.92 (3)	1.07 (4)	0.32 (1)	4.62 (3)	-

T2D^all^ _Type 2 Diabetes (T2D) with or without ischemic stroke (IS); T2D^only^ _T2D patients without IS; T2D^IS^ _T2D patients with IS; IS^only^ _IS patients with no T2D.

P value *<0.05, **< 0.01.

### Disease association between mtDNA polymorphism and disease groups

Odds Ratios (OR) obtained from logistic regression analysis (using adjustment for age, gender, population and haplogroups) between all groups are shown in Table 2 (left column). A male gender effect (Table 2 and Additional file 3: Table S3) showed only in disease groups that included patients with IS history (T2D^all^, T2D^IS^ and IS^only^). The association was most significant in the IS^only^ group (P < 0.001) where age was also a contributing factor. Interestingly, no mtDNA associations were seen in the IS^only^ group. Further, significant associations of haplogroups B5a2b, E, and E2b1 (P < 0.05) were seen in the T2D^only^ group, and of haplogroups B4a1a and B4c1b2 (p < 0.01) in the T2D^IS^ group. Finally, haplogroup D4 showed protection against T2D (OR 0.35, 95% CI 0.18 ~ 0.66 P < 0.001).

**Table 2:** **Significant Odds Ratio (OR, 95**% **CI) between Control and Disease groups using genders, age and mtDNA polymorphism**

**Disease groups**	**Explanatory variables**	**Logistic regression**	**Fisher Exact test using SNPs between control and disease groups**	
		**Gender, age, population and haplogroups (Control = 327 Desease = 481)**	**SNP markers from complete sequencing (Control= 73, T2D**^**all**^**=103, T2D**^**only**^**=70, T2D**^**IS**^**=33, IS= 63)**	**SNP markers from partial sequencing (Control= 327, Disease= 481)**
T2D With and without IS (T2D^all^)	Gender	0.67, 0.51~0.89**	0.40, 0.21~0.74**	0.61, 0.48~0.78***
	Population	1.7, 1.23~2.2***	-	1.7, 1.27~2.31***
	B4a1a	-	3.93, 1.09~14.12*	-
	B5a2b	-	-	2.13, 1.07~4.24*
	D	0.53, 0.35~0.82**	-	0.42, 0.24~0.74**
	D4	0.35, 0.18~0.66***	-	-
	E1	-	-	1.95, 1.04~3.65*
	F4b	-	-	2.56, 1.23~5.34*
T2D^only^	Population	1.5, 1.1~2.0**	2.71, 1.37~5.49**	1.86, 1.36~2.55***
	B4a1a	-	3.83, 1.01~14.54*	-
	B5a2b	2.95, 1.23~7.05*	-	2.38, 1.18~4.79*
	D	0.57, 0.37~0.90*	-	-
	D4*	0.36, 0.18~0.72*	0.25, 0.07~0.93*	0.32, 0.17~0.61***
	D5/M7	-	-	0.57, 0.33~0.98*
	E	1.89, 1.15~3.10*	3.64, 1.25~10.64*	1.86, 1.16~2.97**
	E1a1	-	2.99, 1.09~8.22*	1.86, 1.16~2.97**
	E2b1	4.32, 1.36~13.72*	-	1.86, 1.16~2.97**
	F4b	-	-	1.82, 1.03~3.22*
T2D with IS (T2D^IS^)	Gender	0.52, 0.29~0.91*	0.38, 0.16~0.89*	0.44, 0.28~0.69***
	Age	1.06, 1.03~1.08***	nt	nt
	B4a1a	3.90, 1.70~8.06***	5.19, 1.21~22.22*	2.34, 1.04~5.30*,
	B4c1b2	4.39, 1.50~12.80**	-	-
Ischemic Stroke (IS^only^)	Gender	0.51, 0.32~0.81**	0.29, 0.14~0.58***	0.48, 0.32~0.71***
	age	1.10, 1.08~1.13***	nt	nt
	F4b	-	-	3.09, 1.11~8.59*

T2D^all^ _Type 2 Diabetes (T2D) with or without ischemic stroke (IS); T2D^only^ _T2D patient without IS; and T2D^IS^ _T2D patient with IS; IS^only^ _IS patient with no T2D.

SNPs obtained from partial sequencing include HVS1, np 8000 to np 9000 and np 9800 to np 10900 regions.

P value * <0.05, ** < 0.01, *** < 0.001, nt: not tested.

Odds Ratios analyses with Fisher’s exact test were carried out on a smaller data set of complete sequence of mtDNA genomes (N = 239) (Table 2, middle column).

Most mtDNA-SNPs showing significance correlated with results obtained with the logistic regression analysis (Additional file 4: Table S4: significant mtDNA SNPs associated with diseases). In each set of significant SNPs (column 2 or 3, Table 2 and Additional file 4: Table S4), one SNP was actually a founding position in the mtDNA phylogeny [13] and correlated to findings previously identified using logistic regression. For example, nucleotide positions (nps) 4491, 7598 and 13626 are all key nucleotide determinants of macro-haplogroup E, and nps 4883 and 5178A are key determinants of macro-haplogroup D.

On the contrary, while haplogroup E2b1 showed significance in the logistic regression analysis, nps 3027, 3705, 6620, 10834, 13254, 14577 and 14766 belonged to a different branch determining haplogroup E1a1, a sister branch of E2b1 [13], both branches having in common nps 4491, 7598 and 13626. Such discrepancies between columns in Table 2 were most likely due to sub-sampling when reanalyzing the data using different typing/testing method.

MtDNA partial sequencing results (Table 2 and Additional file 4: Table S4, column 3) were also used as they represented a larger data set (N = 808). It was expected that all significant SNPs would correlate to a haplogroup association determined using logistic regression analysis (B4a1a, B5a2b, D4, and E2b1). As above, few discrepancies were obtained with the two previous analyses. While sampling may be the causal factor, parallel mutations occurring between different haplogroups are commonly seen within the hypervariable control region of the mtDNA and could have contributed to the discrepancy.

Of note, haplogroup F4b was significantly associated with all disease groups when using mtDNA partial sequencing (Table 2 and Additional file 4: Table S4, column 3), it was not selected by logistic regression nor with the complete mtDNA sequencing data set. This result may also be the result of sampling variation and should be used with caution or confirmed with a larger data set.

### Functional and physiological measurements

Comparisons of functional and physiological measurements of the control group with all patients in the four disease groups are shown in Table 3. Lowest levels of LDL and total cholesterol (Table 3) were seen among individuals with T2D^only^ and highest measurements of systolic blood pressure and triglyceride (TG) were seen in the T2D^IS^ group. The IS^only^ group had the lowest TG level and total plasma anti-oxidant capacity (PAO), and the highest oxidation damage of DNA, as inferred from high plasma 8OHdG. In general, these results were as expected from literature review for T2D [14,15].

**Table 3:** t Test, comparison of physical and functional data between control and disease groups

**Physical or functional measurements**	**Control (±2 SD)**	**T2D**^**all**^**(±2 SD)**	**T2D**^**only**^**(±2 SD)**	**T2D**^**IS**^**(±2 SD)**	**IS**^**only**^**(±2 SD)**
	**N = 254**	**N = 271**	**N = 189**	**N = 82**	**N = 132**
BMI (kg/m^2^)	26.1 ± 4.6	26.9 ± 4.9	27.0 ± 5.0	26.8 ± 4.7	25.0 ± 4.5
Systolic BP (mmHg)	130 ± 17	143 ± 26***	136 ± 22*	158 ± 29***	153 ± 24***
Diastolic BP (mmHg)	83 ± 12	78 ± 14**	76 ± 13***	83 ± 16	85 ± 15
HDL (mg/dL)	46 ± 16	38 ± 28	36 ± 14***	42 ± 43	38 ± 14*
LDL (mg/dL)	127 ± 37	107 ± 52*	98 ± 50***	123 ± 52	117 ± 43
Total-Cholesterol: TC (mg/dL)	199 ± 55	174 ± 64***	170 ± 67***	185 ± 51	187 ± 127
Triglyceride: TG (mg/dL)	134 ± 131	169 ± 163	169 ± 174	170 ± 138	120 ± 76
Oxidation damage of DNA: 8OHdG (pg/ml)	4584 ± 1528	5862 ± 2973**	5891 ± 2678**	5799 ± 3566	6894 ± 4555*
Plasma anti-oxidant capacity: PAO (mM)	717 ± 277	746 ± 254	786 ± 268	669 ± 207	635 ± 218
ATP (fmole/cell)	0.64 ± 0.31	0.67 ± 0.34	0.64 ± 0.32	0.73 ± 0.38	0.80 ± 0.45
Hydrogen peroxide production (DCF)	44 ± 38	44 ± 38	46 ± 35	40 ± 45	40 ± 42
Lipid Peroxidation	1.25 ± 1.45	1.07 ± 1.05	1.11 ± 0.98	0.95 ± 1.22	1.73 ± 5.75
Mitochondrial membrane potential (DiOC)	15 ± 10	13 ± 9	13 ± 8	14 ± 11	13 ± 14

P value: *< 0.05, **< 0.01,***< 0.001.

Pairwise comparisons of physiological and mitochondrial functional measurements between control and disease groups within haplogroups that were determined as significant in Table 2 (Additional file 5: Table S5) showed little variation within groups. However when comparing specific measurements between the disease groups, variation was noticeable. This suggested that specific mtDNA haplogroups/haplotypes could affect the outcome of functional and physiological measurements, and possibly reflect the health condition of the individuals.

**Figure 1:**
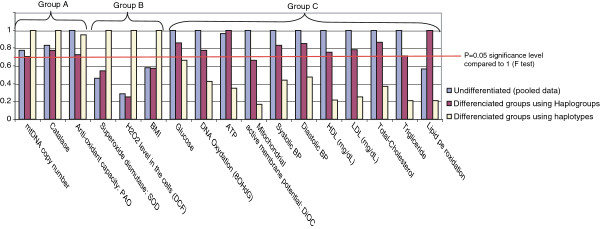
**F Test: Normalized variance of measurements between the pooled Data and groups differentiated according to Haplogroups and Haplotypes.** Clinical measurements and their variance (F test).

To determine if the mtDNA polymorphism affect the health status of the individuals, the variances of physiological and functional measurements of the pooled data set were compared with the data regrouped according to haplogroups and haplotypes (Figure 1). The complete mtDNA genomic sequence data set (73 controls, 71 T2D^only^, 33 T2D^IS^ and 63 IS^only^) was used and stratified into test specific triplets each containing a subset of the test regrouped according to haplogroup (red), a subsets regrouped according to haplotypes (white) and a pool of the two subsets (blue). In Figure 1, each variance in a triplet was divided by the highest variance obtained in that triplet hence normalizing the data into a zero to one scale of ratios. It was hypothesized that the variances in each test specific triplets would show little differences to its pooled variance if the mitochondrial DNA polymorphism had no effect on the disease state (i.e. of T2D) of the individuals. No significant variance changes were seen for mitochondria copy number, Catalase and anti-oxidant capacity (PAO). Alternatively, a mitochondrial genetic effect on the health status (i.e. of T2D) should be associated with a significant amount of variance difference between the pooled data and the subsets in a triplet. The higher polymorphism seen in the pooled data groups of the 11 triplets on the right of Figure 1 showed higher variance (P < 0.05) than seen in the corresponding haplotype subsets, indicating a mitochondrial genetic effect on the health status. The unexpected patterns seen with triplets for superoxide dismutase, H_2_O_2_ intracelular level and body mass index showing a reversed relationship (with haplotype variance being much larger than the pooled data) were not expected.

### Non-synonymous mutation np 15746 in B4a1a may affect the health status

MtDNA haplogroup B4a1a is prevalent in Taitung (southern Taiwan). The mtDNA phylogeny determines this haplogroup with one control region variation (np 16519) and three coding region variations (nps 6719, 12239 and 15746). Only transition A15746G corresponds to a change of Isoleucine (Ile) to Valine (Val) at amino acid position 334 in cytochrome b. This mutation was first reported in a T2D Japanese individual bearing mtDNA haplogroup G (Human Mitochondrial Genome Polymorphism Database, http://mtsnp.tmig.or.jp/mtsnp/index_e.shtml, NDsq0208). A search of the Phylotree database [13] also showed the presence of this mutation in haplogroup N8. This indicates that the association of np 15746 with T2D may be seen with different haplogroups in different populations (G3a in Japan, B4a1a1 in Island Southeast Asia and putatively N8 in South China) [16,17]. Accordingly, we constructed the inferred global structures of the Cytochrome b protein of B4a1a, G3a, and N8 individual using the I-TASSER protein structure prediction algorithm [18]. Amino acid 334 of Cytochrome b appeared in close proximity to the ubiquinol binding site at amino acid 278 [19], in the 3D structure (data not shown), and a force field energy increase from −39 KJ/mole to −7 KJ/mole [20] suggested reduction of the stability of the Cytochrome b coenzyme Qo pocket (Additional file 6: Table S6). The potential to affect the protein function of cytochrome b in carriers of variant 15746 was equally confirmed using the structure based method I-Mutant v2.0 software [21]. Further, when using the 3D protein structure prediction modeling program RaptorX [22] for Cytochrome b, the Ile to Val amino acid switch at 334 appeared to affect the highly conserved “PEWY” motif (Amino Acid position 270–273) within the Qo pocket [19] with a force field energy change at Amino Acid 273 (Tyr) increasing from −10 to 725 KJ/mole and again suggesting reduction of Qo activity (Additional file 6: Table S6).

Finally, a note of caution comes to order as the average younger age of the control group (Additional file 3: Table S3) and the high CV of flow cytometry test results (Additional file 2: Table S2) may influence the outcome of the statistical tests of this study.

## Discussion

Several mtDNA disease association studies have been reported on

i) mtDNA mutations related to maternal inherited diseases, such as MELAS, LHON, MERRF [23];

ii) mtDNA haplogroup association to disease occurrence [6-8,24-28]; and

iii) approaches using animal models to study mtDNA mutations in disease and aging [29,30].

All these studies have ubiquitously agreed on the significant importance of the role of the mitochondria on the health status of the individual. Our study used partial and complete mtDNA genome sequencing to first determining which mtDNA haplogroup was associated with the occurrence of T2D among southern Taiwanese (Tables 1, 2 and 3), and secondly determine if functional or physiological measurements in T2D probands showed variation with different mtDNA polymorphism (Figure 1).

### Mitochondrial polymorphism influence physiological and mitochondrial functional measurements

All tissues except erythrocytes carry mitochondria. Mitochondria are the main site of formation of reactive oxygen species (ROS) byproducts in the cells. ROS can modify the function of many essential signal transduction pathway components and transcription factors [31,32]. While having an effect on cells apoptosis it also has a recruiting effect on platelets and leukocytes to induce the host defense [33]. The relationship mtDNA polymorphism, ROS and its effect on the general physiological performance is not fully understood. In animal model, researchers have observed that variation in the count of mtDNA SNPs was associated with hypertension [34] and to ROS activity. On the other hand it was observed that suppression or reduction of ROS could be beneficial to endothelial cell relaxation [35] and could lower blood pressure. Further, lower efficiency of the electron transport chain (ETC.) was associated to ROS induced morbidity, and malfunction of the mitochondrion associated with hyperlipidemia [36,37]. In the current study, only mtDNA haplogroup B4a1a in the T2D^IS^ group showed significant variation in systolic blood pressure (Additional file 5: Table S5). This observation was in agreement with cybrid studies analyzing mtDNA haplogroup associations with different cellular biochemical functions [38,39]. The comparisons of variance (Figure 1) between groups stratified according to pooled data, haplogroups and haplotypes confirmed these findings suggesting that groups with lowest mtDNA diversity (haplotype groups) produced the lowest variance between functional measurements. This was shown in Figure 1 for ATP production, lipid peroxidation, DNA oxidation, mitochondrial inner membrane potential, lipid metabolism (LDL, HDL, total cholesterol, and triglyceride) and blood pressure. In summary, mtDNA variation may influence the activity of the protein in the mitochondrial electron transport chain complexes (ETC.) which is the major generators of ROS in cells and tissues.

### Nucleotide 15746 of mtDNA haplogroup B4a1a is a risk factor for T2D in Island Southeast Asia and Oceania

Probands bearers of mtDNA haplogroup B4a1a, a sub-haplogroup of B4, had higher systolic BP (P < 0.01), DNA oxidation (8OHdG) levels and lower serum triglyceride. These measurements had large SD when compared with the control group (Table 3). However, when only selecting healthy individuals with haplogroups ancestral to B4a1a (i.e. individuals with B4, B4a, B4a1), the SD remained the same as the control group (data not shown). This suggested that only variant nucleotides np 6719, 12239, 15746 and 16519 that determine the more recent B4a1a branch in the B4 phylogeny was associated with T2D.

The A to G transition at np 15746 is the only non-synonymous mutation on this branch and corresponds to a change of amino acid 334 in cytochrome b from isoleucine (Ile) to valine (Val). The same transition (A15746G) was also observed in one Japanese T2D patient with mtDNA haplogroup G, and with low frequency in southern Chinese haplogroup N8. When using the I-TASSER or Raptor X 3D protein structure modeling software, valine 334 was located in close proximity to the coenzyme Q binding site (amino acid 278 of Cytochrome b) and strong force field energy changes were predicted near or within the Q pocket by both models. Amino acids 271 (GLU), 273 (TYR) and 278 (TYR) of human Cytochrome b are important in the ubiquinol oxidation and binding of the Rieske protein for the electrons transport of complex III [40-42]. The 3D protein structure models inferred using I-TASSER and Raptor X 3D algorithms suggested instability and lower efficiency of Cytochrome_b Q_o_ site function. It is therefore plausible that the reactive oxygen species (ROS) and H^+^ movements from the mitochondrial matrix to the intermembrane space may diminish the respiratory chain enzyme efficiency [41] in B4a1a individuals. Haplogroup B4a1a and its derived sub-haplogroups (B4a1a1, B4a1a1a) are all determined by np 15746, and are prominent in Island Southeast Asia and Oceania [3,43-45]. Actually, the occurrence of T2D in ISEA, Oceania and the Pacific Region is very high (WTO STEPS Country Reports: American Samoa NCD Risk Factors; http://www.who.int/chp/steps/Printed_STEPS_Report_American_Samoa.pdf). It was shown that the prevalence of T2D in the 55 to 64 years age group in Samoa is 71%. Also the major causes of death, coronary heart disease, stroke, high blood pressure and mature onset diabetes are all associated to obesity (WTO STEPS Country Reports: American Samoa NCD Risk Factors; http://www.who.int/chp/steps/Printed_STEPS_Report_American_Samoa.pdf). Further the frequency of B4a1a1a in Samoa in ~80% [43]. This health pattern is surprisingly similar to the one seen among carriers of haplogroup B4a1a in our Taitung data set in southern Taiwan and further support the argument that np 15746 could be a risk factor associated to T2D occurrence. Of note, SNP 15746 carriers with other mtDNA haplogroup have never been seen in Taiwan.

It has been postulated that the prominence of np 15746 in ISEA, Taiwan and Polynesia may be the result of an evolutionary advantage such as adaptation to the hot and humid environment of these regions [46]. It was recently hypothesized that insulin resistance may render defender cells more immuno-efficient by having better access to energy [5]. How an evolutionary survival advantage can be associated to B4a1a and at the same time lower the health status of the individual still remains matter to further studies.

## Conclusions

Regardless of age, men seemed generally more susceptible to T2D than women in southern Taiwan. Although the significant association of B4a1a and E2b1 with T2D ceased when corrected for multiple test, these haplogroups are seen only among Taiwan Aborigines, Island Southeast Asians and Pacific Ocean islanders where T2D is predominant. More over, we showed that specific mtDNA genetic profiles may produce specific physiological and biochemical measurements outcomes. More understanding of the function of the mitochondrion and its relation to T2D will indicate ways to the clinician of influencing the early course of the disease.

### Accession numbers

The GenBank accession numbers (http://www.ncbi.nlm.nih.gov/nucleotide/) for HVS-1data in this article are as follows: HVS-1 (KC251766-KC252334). Complete sequence data: accession numbers (KC252335-KC252573).

## Abbreviations

mtDNA: Mitochondrial DNA; T2D: Type 2 diabetes; IS: Ischemic stroke; HG: Haplogroup; ISEA: Island Southeast Asia; HVS-1: Mitochondrial DNA hypervariable region 1; np: Nucleotide position; T2Donly: T2D patients with previous T2D history, but no IS history; T2DIS: T2D patients having previous T2D and IS histories; ISonly: Patients with previous IS history but no T2D history.

## Competing interests

The authors declare that they have no competing interests.

## Authors’ contributions

ML, JHL and JT conceived and designed the study. KNH, KHH, YH, and WMN collected controls and patients’ sample. JHL, JCY, ZSC and CYH performed mtDNA sequence and mitochondrial function analysis. YHW instructed mitochondrial function analysis. JHL and JAT performed statistical analyses. JHL and JAT wrote the paper. All authors read and approved the final manuscript.

## Supplementary Material

Additional file 1: Table S1 Patient description. Click here for file

Additional file 2: Table S2 Coefficient of variation of mitochondrial functional tests. Click here for file

Additional file 3: Table S3 Mean age demorgraphy in control and disease groups. Click here for file

Additional file 4: Table S4 Significant Odds Ratio between Control and Disease groups using genders, age and mtDNA polymorphism. Click here for file

Additional file 5: Table S5 t Test, comparison of physical and functional measurements between control and disease groups using haplogroups determined significant in Table 1 and 2. Click here for file

Additional file 6: Table S6 3D protein structure. Inferred Force Field Energy of the Qo site in cytochrome b using I-TASSER and Raptor X models. Click here for file
